# Progesterone reverses the mesenchymal phenotypes of basal phenotype breast cancer cells via a membrane progesterone receptor mediated pathway

**DOI:** 10.1186/bcr2588

**Published:** 2010-06-11

**Authors:** Lian Zuo, Wei Li, Shaojin You

**Affiliations:** 1 Atlanta Research & Educational Foundation (151F), Atlanta VA Medical Center, 1670 Clairmont Road, Decatur, GA 30033, USA

## Abstract

**Introduction:**

Basal phenotype breast cancers (BPBC) are often associated with apparent epithelial to mesenchymal transition (EMT). The role of progesterone (P4) in regulating EMT of BPBC has not been reported.

**Methods:**

The EMT relevant biology was investigated *in vitro *using human BPBC cell models (MDA-MB468 and MDA-MB231) with P4, PR agonist (RU486), and PR antagonist (R5020) treatments. The essential role of membrane progesterone receptor α (mPRα) in the P4-regulated EMT was demonstrated by knocking down the endogenous gene and/or stably transfecting exogenous mPRα gene in the BPBC cell models.

**Results:**

The expression of snail and down-stream EMT proteins such as occludin, fibronectin, and E-cadherin was significantly regulated by P4 incubation, which was accompanied by cell morphological reversion from mesenchymal to epithelial phenotypes. In searching for the cell mediator of P4' action in the MDA-MB468 (MB468) cells, it was found that mPRα but not the nuclear PR has an essential role in the P4 mediated EMT inhibition. Knocking down the expression of mPRα with specific siRNA blocked the P4's effects on expression of the EMT proteins. In another BPBC cell line - MDA-MB231 (MB231), which is mPRα negative by Western blotting, P4 treatment did not alter cell proliferation and EMT protein expressions. Introduction of the exogenous mPRα cDNA into these cells caused cell proliferation, but not EMT, to become responsive to P4 treatment. In further studies, it was found that activation of the PI3K/Akt pathway is necessary for the P4-induced EMT reversion. To define the potential inter-mediate steps between mPRα and PI3K, we demonstrated that mPRα, caveolin-1 (Cav-1), and epidermal growth factor receptor (EGFR) are colocalized in the membrane of caveolar vesicle and the P4-repressed EMT in MB468 cells can be blocked by EGFR inhibitor (AG1478) and PI3K inhibitor (wortmannin).

**Conclusions:**

Our data suggest that the signaling cascade of P4 induced mesenchymal repression is mediated through mPRα and other caveolae bound signaling molecules namely Cav-1, EGFR, and PI3K. This novel finding may have great impact on fully understanding the pathogenesis of BPBC and provide an essential clue for developing a targeted therapeutic strategy for treatment of BPBC.

## Introduction

Basal phenotype breast cancer (BPBC) is a subtype of cancer with apparent mesenchymal phenotypes. Boyer and colleagues first described a morphologic change from epithelial-like sheets of cultured cancer cells to scattered, fibroblast-like cells capable of invading the basement membrane, so called epithelial to mesenchymal transition (EMT) [1]. The morphologic criteria of EMT *in vitro *involve changes in cell polarity, separation into individual cells and acquisition of cell motility [2]. These changes can be either stable or reversible. The essential changes in gene expression that disrupt cell polarity and cause mesenchymal transition have been identified. Snail, twist, and slug have been shown as key regulators of EMT in both animal and human cancers [3]. Among these genes, snail acts as a transcriptional factor to repress genes that encode the cell-cell junctional apparatus, such as E-cadherin and occludin; and to enhance genes that encode mesenchymal or tumor interstitial components, such as fibronectin and vimentin, resulting in a dedifferentiated mesenchymal transition characterized by increased cell motility [4,5].

The roles of female sex hormones such as progesterone (P4) in the pathogenesis of BPBC remain unclear. Classically, the actions of P4 on cancer cells are attributed to the binding of nuclear progesterone receptor (PR), translocation of P4/PR complex into the nucleus and subsequent activation of target genes over the course of several hours. These mechanisms, however, are not applicable to BPBC due to a lack or very low level of PR expression in these cancers. The mechanisms for P4's actions in modulating the cancer biology of BPBC remain largely unknown. Recently, the cell membrane hormonal receptors, such as membrane progesterone receptor (mPR) family and progestin membrane receptor component 1 (PGMRC1), were identified and demonstrated functional in BPBC [6,7]. It is believed that the rapid responses of P4 are initiated at the cell surface by binding to the membrane receptors [8-10]. For examples, progestin, a synthetic P4, has been shown to activate a variety of signaling pathways through mPRα [6]. The binding of progestin to mPRα alters the secondary messenger pathways through activation of the pertussis toxin-sensitive inhibitory G-proteins and then activates the mitogen activated protein kinases (MAPK)/Erk 1/2 pathway [6,7,11,12]. However, this theory has been debated because others failed to demonstrate mPRs on the cell surface or mediate P4-dependent signaling events, such as coupling to G proteins [13]. Moreover, mPRs were shown to be primarily situated in the endoplasmic reticulum [13,14]. In this study, we co-localized mPRα, caveolin-1 (Cav-1), and epidermal growth factor receptor (EGFR) at a specified membrane structure, so called caveolar vesicle, and demonstrated that P4 reverses the mesenchymal phenotypes of human BPBC cells (MB468 and MB231) via a caveolae bound signaling complex namely mPRα, Cav-1, EGFR, and PI3K/Akt. Further study on this unique molecular pathway may afford great potential to discover novel molecular targets for treatment of BPBC.

## Materials and methods

### Chemicals and antibodies

RU486 (mifepristone), AG1498, wortmannin, PP1 and PD98052 were purchased from EMD Chemicals (Gibbstown, NJ, USA); R5020 (promegestone) and bpV (phen) were from PerkinElmer (Waltham, MA, USA) and Thermo Fisher Scientific (Pittsburgh, PA, USA), respectively. Anti-snail antibody was from Abcam (1:1000, Cambridge, MA, USA); anti-E-cadherin and anti-fibronectin antibodies were obtained from EPITMICS (1:1000, Burlingame, CA, USA); anti-mPRα, anti-GAPDH (1:500) and secondary antibodies (1:2000) were purchased from Santa Cruz Biotechnology (Santa Cruz, CA, USA); anti-occludin antibody was from BD transduction (1:500, San Jose, CA, USA); and anti-α-tubulin antibody was from Sigma (1:2000, St. Louis, MO, USA).

### Cell culture

The human breast cancer cell lines MDA-MB468 (MB468), MDA-MB231 (MB231) and human embryonic kidney 293 (HEK 293) cells were obtained from the American Type Culture Collection (Rockville, MD, USA). Both human breast cancer cell lines were negative for estrogen receptor (ER) and human epidermal growth factor receptor (Her)-2 and classified as 'basal phenotype A' cells [15]. The cultured MB468 cells at early passages typically appear like epithelial cells with oval and/or polygonal shapes [16]; and after multiple passages (50+), these cells exhibit apparent mesenchymal phenotypes with spindle and elongated shapes [see Additional file 1], which are ideal for the proposed studies. Long-term cell culture *in vitro *may generate genetic instabilities and the derived cell lines with altered cell biological features have been utilized as cell models for *in vitro *studies [17,18]. The late passage MB468 cells and early passage MB231 cells with apparent mesenchymal phenotypes [16] were cultured and maintained at 37°C with 21% oxygen and 5% carbon dioxide (without 5% carbon dioxide for MB231 and HEK293 culture) in DMEM (Sigma, St. Louis, MO, USA) containing 10% FBS (Gibco, Carlsbad, CA, USA), 2 mM L-glutamine, 100 U/ml penicillin, and 100 μg/ml streptomycin (Gibco, Carlsbad, CA, USA) and maintained in a humidified incubator. The XTT cell proliferation assay kit was from Cayman Chemicals (Ann Arbor, MI, USA).

### Immunoblotting

Western blot assays were performed as described previously [19]. After treatment with or without P4 and/or diverse pathway inhibitors, the growth-arrested cells were lysed with 500 μl ice-cold lysis buffer, pH 7.4 (50 mM HEPES, 5 mM EDTA, 50 mM NaCl), 1% Triton X-100, protease inhibitors (10 μg/ml aprotinin, 1 mM phenylmethylsulfonyl fluoride, 10 μg/ml leupeptin) and phosphatase inhibitors (50 mM sodium fluoride, 1 mM sodium orthovanadate, 10 mM sodium pyrophosphate). Cell lysates (25 μg) were separated using SDS-PAGE and transferred to nitrocellulose membranes, blocked overnight in PBS containing 6% nonfat dry milk and 0.1% Tween 20, and incubated for one hour with primary antibodies at proper dilutions. After incubation with secondary antibodies, proteins were detected by ECL chemiluminescence. Image J was used for quantitative analysis.

### Cell morphological changes of MB468 treated with P4

MB468 cells (10^5 ^cells/dish) were seeded and grown in 35 mm cell culture dishes (Bioptechs Inc., Butler, PA, USA) for 24 hours. The medium was changed to complete culture medium with or without 30 ng/ml of P4 for 48 hours and then cultured as indicated. Nomarski differential interference contrast (DIC) images were taken using a confocal microscopy (Olympus FV1000, Tokyo, Japan) with a transmitted light at 400× magnification.

### Cell proliferation assay

The XTT cell proliferation assay was performed according to the manufacturer's protocol. Briefly, cells were seeded in a 96-well plate in 100 μl of culture medium with or without the compounds to be tested and incubated for 24 to 48 hours at 37°C. The reconstituted XTT mixture (10 μl/well) was added and the cells were incubated for two hours. The absorbance of each sample was subsequently measured using a microplate reader at a wavelength of 450 nm.

### Knocking down mPRα expression with small interference RNA

Cells were transfected with mPRα small interference RNA (siRNA) or an equal amount of nonspecific control siRNA (Dharmacon, Lafayette, CO, USA) using the Oligofectamine reagents according to the manufacturer's protocol (Invitrogen, Carlsbad, CA, USA). Two days after transfection with siRNA, the cells were incubated with diverse experimental reagents.

### Transfection of mPRα DNA plasmid

The MB231 cells were cultured and split when the cell confluence reached about 90%. The human mPRα cDNA constructed in a pUC-based plasmid with CMV promoter (pBK-CMV) vector [20] was purified and then transfected into the cells using Lipofectamine 2000 reagent following the manufacturer's instructions (Invitrogen, Carlsbad, CA, USA). Two days after transfection, the mPRα expressing cells were selected with 1000 μg/ml G418 (Gibco, Carlsbad, CA, USA). The resistant colonies were then isolated and propagated with 500 μg/ml G418 in order to produce the stably transfected cell lines.

### Isolation of caveolar fractions

Caveolae membranes were isolated as described previously [21]. Briefly, MB468 cells was homogenized in 1 ml of 2-(*N*-morpholino) ethanesulfonic acid (MES)-buffered saline (24 mM MES, pH 6.5, and 0.15 NaCl) plus 1% Triton X-100 and spun down at 3,000 *g *for five minutes at 4°C. The supernatant (4 ml) was used to dissolve sucrose and compose 40% of sucrose solution. This solution was placed in a 12.5-ml Beckman centrifuge tube (Beckman Coulter, Fullerton, CA, USA) with a 5 to 30% sucrose gradient layered on top and then centrafuged at 39,000 rpm for 24 hours at 4°C in a Beckman SW-41 rotor. After the centrafuge, 600 μl fractions of the solution were collected and subjected to further analysis.

### Immunohistochemical analysis

In brief, two tissue microarray slides consisting of human breast cancer (140 and 70) cores and adjacent benign breast tissue (10 and 24) cores were purchased from the Biomax US (Rockville, MD, USA). These tissue microarray slides were constructed with complete different sets of tissue blocks. There were total 105 breast cancer and 17 benign breast tissues in these two tissue arrays. Two 1.5 mm-cores from each breast tissue block were constructed in the tissue microarray slides. After deparaffinization, rehydration, antigen retrieval, and endogenous peroxidase blocking, the slides were blocked with 5% normal horse serum for one hour and sequentially incubated with anti-mPRα antibody (1:200 dilution) at 4°C overnight and then incubated with a secondary antibody at room temperature (see manual of the ImmPRESS REAGENT kit, VECTOR Lab, CA). The color was developed with the ImmPACT DAB kit (VECTOR Lab, Burlingame, CA, USA). Between the incubations, the slides were washed twice with 1× PBS buffer (5 minutes each) [19,22]. Two negative controls were included: (1) control slides were stained without the primary antibody; (2) control slides were incubated with a specific blocking peptide (cat# sc-50111p, Santa Cruz, CA, USA) prior to the primary antibody incubations. The immunostained slides were counterstained with hematoxylin and evaluated using a Nikon microscope with an Olympus digital camera (Tokyo, Japan). The immunohistochemical result was evaluated using a semi-quantitative scoring system by a trained research pathologist, who was blinded to patients' clinical data provided by the company. The intensity of the immunostaining was defined into four categories (strong positive, moderate, weak, and negative) [see Additional file 2].

### Statistical analysis

The quantitative data was expressed as mean ± standard error and statistical significance was assessed by Student's paired two-tailed t-test. The positive rates of mPRα immunostains in different groups of human breast cancers and benign diseases was compared and analyzed by Fisher's exact test. *P *< 0.05 was considered significant.

## Results

### P4 regulates expression of snail and other EMT-relevant proteins in MB468 but not in MB231 cells

**Figure 1:**
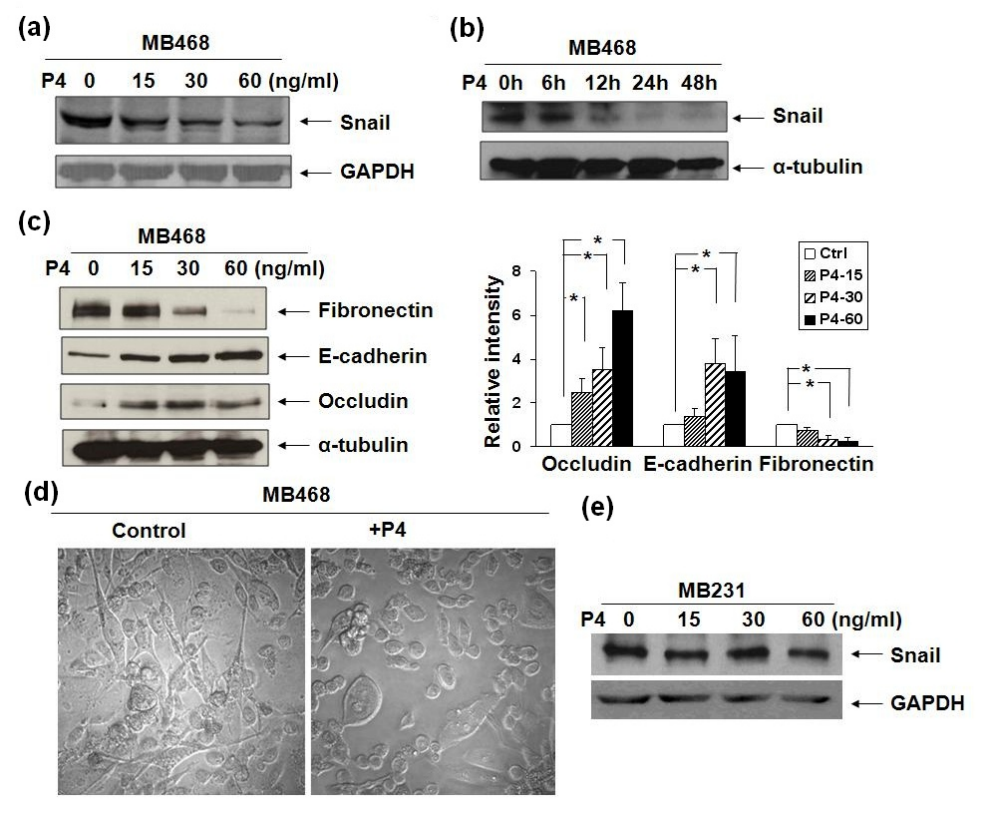
**Dose curve and time course of P4's action on epithelial-mesenchymal transition relevant events of MB468 and MB231 cells**. The growth-arrested MB468 cells were treated with different doses of progesterone (P4) for **(a and c) **24 hours or with P4 at **(b) **30 ng/ml for various times as indicated. Western blot analyses were performed with diverse antibodies as indicated. The expression of snail was inhibited by P4 treatments in dose- and time-dependent manners (Figures 1a and b); and the expression of fibronectin, E-cadherin, and occludin was also modulated by P4 in dose dependent manners. **(d) **Morphological changes of MB468 cells treated with/without P4 at 30 ng/ml for 48 hours. Photos (DIC images) were taken by using confocal microscopy at 400× magnification. **(e) **The growth-arrested MB231 cells were treated with different doses of P4 for 24 hours as indicated. Western blot analyses were performed with anti-snail and anti-GAPDH antibodies. Equivalent levels of snail expression were shown after the P4 treatment at different doses as indicated. All data were collected and averaged from three individual experiments and the graphs are expressed as fold change over basal. * *P *< 0.05 for difference of protein expression induced by P4 vs. vehicle alone.

**Figure 2:**
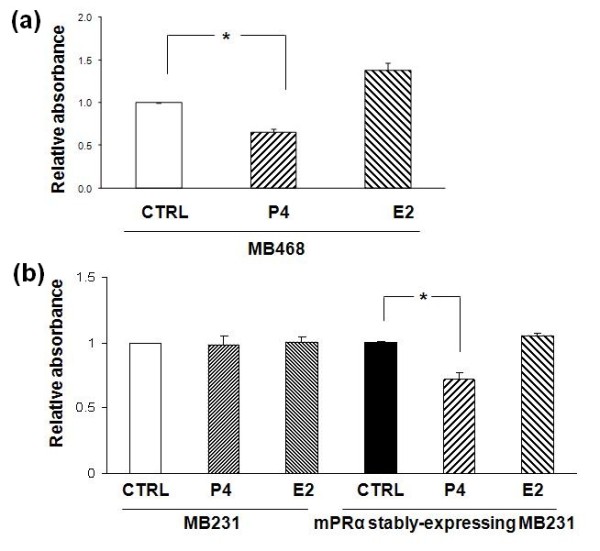
**Effects of P4 on cell proliferation of (a) MB468, parent MB231 and mPRα stably-expressing (b) MB231 cells**. The data are representative for three individual experiments and the graphs are expressed as fold change over the basal. The cells treated by E2 (30 pg/ml for 24 hours) and vehicle alone were used as controls. * *P *< 0.05 for difference of cell proliferation induced by progesterone (P4) vs. vehicle alone.

In this study, we focused on the effects of P4 on expression of snail and other EMT marker proteins. As shown in the Figures 1a and 1b, the snail expression in the late passage MB468 cells was down-regulated by P4 treatment in a dose-dependent (P4 at 15 ng/ml - 47.0 ± 7.4%; 30 ng/ml - 68.3 ± 6.7%; 60 ng/ml - 86.3 ± 1.6%; Figure 1a) and time-dependent manners (6 hours - 20.0 ± 2.4%; 12 hours - 24.0 ± 3%; 24 hours - 87.0 ± 7.4%; 48 hrs - 94 ± 8.6%; Figure 1b). As snail has been known as a key modulator for EMT [4,5], the P4-induced EMT relevant changes were further investigated. As shown in Figure 1c, fibronectin expression was significantly inhibited around 74.0 ± 3.7% by P4 treatment at 30 ng/ml while expression of E-cadherin and occludin was up-regulated about 3.8 fold and 3.5 fold, respectively. Furthermore, the changes in expression of snail/other EMT-relevant proteins were followed by cell morphological changes. The late passage MB468 cells without P4 exposure showed apparent mesenchymal phenotypes, characterized by diverse sizes and spindle or elongated shapes are usually shown; while with P4 treatment, most of the cells appeared epithelial-like transition, featured by large and polygonal shapes or small oval shapes (Figure 1d at 40×) [see Additional file 3]. In addition, the cell proliferation of MB468 was also inhibited by P4. As shown in Additional file 4, the growth of MB468 cells was inhibited by P4 treatments in a dose-dependent manner (15 ng/ml - 19%, 30 ng/ml - 29.5%, and 60 ng/ml - 52.3%, respectively), which is consistent with the previous report [23]. Interestingly, in the early passage MB231 cells, another BPBC cell line with obvious mesenchymal features, the P4 treatment had no effect on snail expression (Figure 1e) and cell proliferation (data in Figure 2b).

### The nuclear PR has no roles on the P4-repressed EMT in MB468 cells

**Figure 3:**
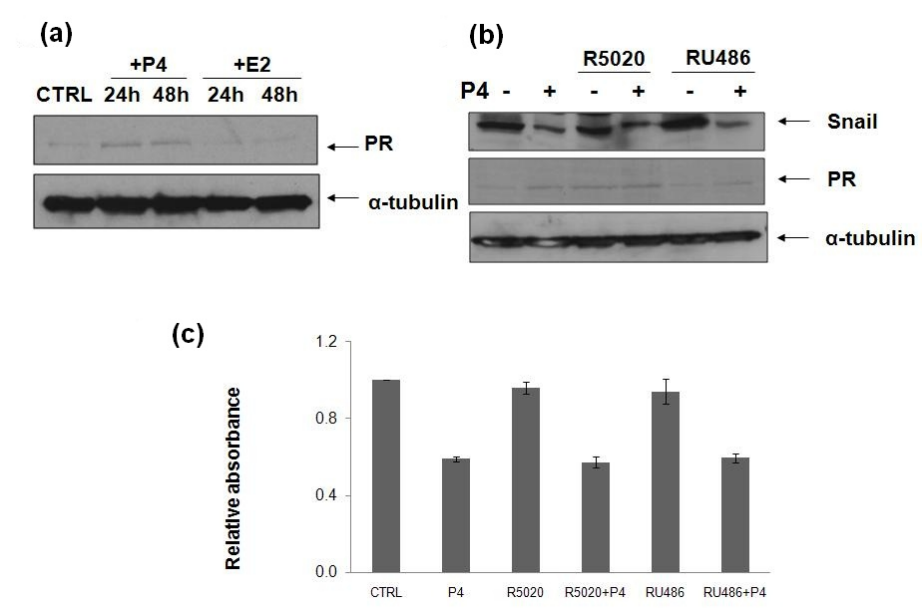
**The role of PR in the P4-repressed epithelial-mesenchymal transition and cell proliferation**. **(a) **Growth-arrested MB468 cells were treated with progesterone (P4; 30 ng/ml) or E2 (30 pg/ml) for 24 and 48 hours as indicated. **(b) **Growth-arrested MB468 cells were treated with R5020 (10 nM) or RU486 (10 nM) for two hours, followed by P4 treatment at 30 ng/ml for 24 hours. Western blot analyses were performed with anti-snail, anti-progesterone receptor (PR) and anti-α-tubulin antibodies. **(c) **MB468 cells were treated with R5020 (10 nM) or RU486 (10 nM) for two hours, followed by P4 treatment at 30 ng/ml for 24 hours. Cell proliferation assays were then performed. As shown in the Figures 3a to c, the P4 treatment inhibited snail expression and cell proliferation at equivalent levels. The results are representative for three separate experiments.

The classical nuclear PR was first considered as a molecular mediator of the P4-repressed EMT in MB468 cells even though they are basically negative for nuclear PR expression in normal culture condition. The cancer progenitor cells, however, may proliferate and express PR in response to sex hormone treatments [24]. As shown in Figure 3a, MB468 cells were treated by P4 and estrogen (E2) and the expression of PR was slightly induced by P4 treatment, but not by E2. To explore whether the increase of PR expression is involved in the P4-repressed EMT events, MB468 cells were then co-incubated with P4 plus RU486 (mifepristone) or R5020 (promegestone), which are known as a PR-specific blocker or agonist. Surprisingly, both PR modulators had no effects on the P4 repressed snail expression (Figure 3b) and cell proliferation (Figure 3c), suggesting that other molecular mediators, but not nuclear PR, might be involved in the P4 repressed EMT events.

### MPRα plays an essential role in the P4-repressed EMT in MB468 cells

**Figure 4:**
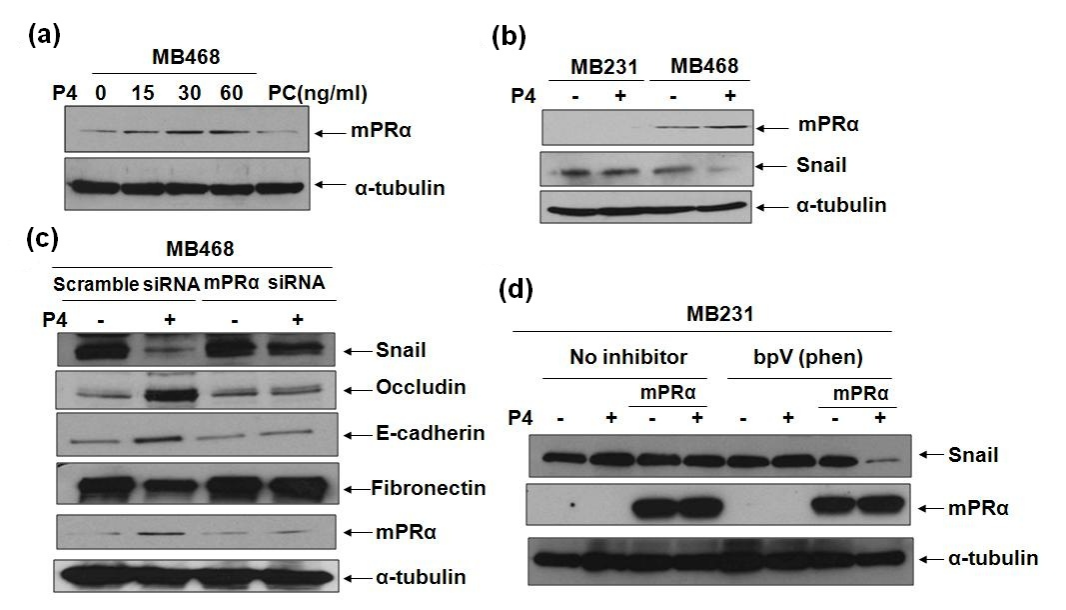
**The role of mPRα in the P4-repressed EMT of MB468 and MB231 cells**. **(a) **The growth-arrested MB468 cells were treated with diverse concentrations of progesterone (P4) for 24 hours. (PC: HEK293 cell lysate as a positive control for membrane progesterone receptor (mPR) α expression.) **(b) **MB231 cells and MB468 cells were treated with/without P4 at 30 ng/ml for 24 hours. Western blot analyses were performed with anti-mPRα, anti-snail, and anti-α-tubulin antibodies. **(c) **MB468 cells were transfected with 50 nM of mPRα siRNA and 50 nM of scramble siRNA; and then treated with P4 at 30 ng/ml for 24 hours. The Western blot analyses showed similar patterns of snail/epithelial-mesenchymal transition (EMT) protein expressions in the cells transfected with scramble siRNA and equivalent levels of snail/EMT proteins in the cells transfected by mPRα siRNA, indicating the roles of specifically knocking down mPRα in MB231 cells. **(d) **The parent MB231 and mPRα stably-expressing MB231 cells were treated with/without bpV(phen) at 1 μM for one hour, followed by P4 treatment at 30 ng/ml. Western blot analyses were performed with anti-snail, anti-mPRα, and anti-α-tubulin antibodies. The data are representative for three individual experiments.

Within the past few years, evidence has been obtained for the involvement of mPRα in the P4's actions in a variety of cell types [6,7,11,12]. In the present study, the expression of mPRα in MB468 cells was up-regulated by P4 treatments in dose-dependent manners (15 ng/ml - 1.97-fold, 30 ng/ml - 2.75-fold, and 60 ng/ml - 2.92-fold, respectively; Figure 4a). As a control, there were no detectable mPRα protein found in MB231 cells (Figure 4b), which is consistent with a previous report [20]. These data suggest a potential role of mPRα in the P4 signaling of BPBC cells. To further demonstrate our hypothesis, the expression of mPRα in MB468 cells was knocked down by mPRα-specific siRNA. As shown in Additional file 5, mPRα siRNA specifically inhibited mPRα expression without affecting α-tubulin expression. After transfection with 50 nM of mPRα siRNA, the P4's effects on the EMT marker proteins were significantly inhibited (Figure 4c).

### Activation of PI3K/Akt is necessary for P4's action on EMT but not on cell proliferation

To further prove the involvement of mPRα in P4's action on human BPBC cells, the mPRα-expressing plasmid DNA was introduced into the parent MB231 cells and then treated by P4 as indicated. There was no reduction found in the expression of snail as compared with that of parent MB231 cells (Figure 2a). By comparing the molecular profiles of MB468 and MB231 cells, major differences were noticed between the two cell lines in phosphatase and tensin homolog gene (PTEN) expression and PI3K/Akt activation [15]. PTEN is an essential inhibitor for the PI3K/Akt pathway [25]. In MB468 cells, there is no PTEN expression and PI3K/Akt pathway is constantly activated. On the contrary, in MB231 cells PTEN is abundantly expressed and PI3K/Akt pathway is always inactivated. To explore the role of PTEN and PI3K/Akt pathway in the P4 regulated EMT, the mPRα stably-expressing MB231 cells were incubated with the PTEN-specific inhibitor bpV(phen) to transactivate the PI3K/Akt pathway. As shown in Figure 4d, snail expression was clearly down-regulated by P4 treatment about 89.8 ± 1.9%. These data strongly suggest that mPRα plays an important role in the repression of EMT through the activated PI3K/Akt pathway in BPBCs.

To test whether the female sex hormone controls cell proliferation of MB468 cells, we incubated the cells with P4 (30 ng/ml) for 24 hours. As shown in Figure 2a, a 34% reduction in cell proliferation was observed in the MB468 cells with treatment of P4, as compared with the cells with treatment of vehicle alone. As expected, P4 had no effects on cell proliferation of the parental MB231. However, the treatment of the mPRα stably-expressing MB231 cells with P4 induced a significant reduction of cell proliferation (28.1%; Figure 2b). These data suggest that mPRα is also involved in regulating cell proliferation of BPBC cells.

### EGFR and PI3K are involved in the P4-repressed EMT in MB468 cells

**Figure 5:**
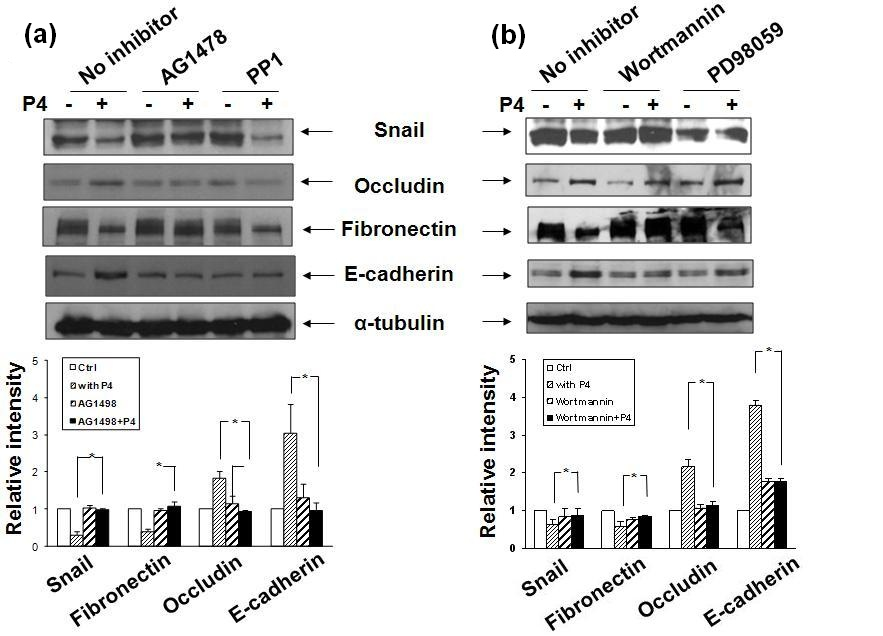
**The molecular pathways involved in the P4-repressed EMT of MB468 cells**. Growth-arrested MB468 cells were treated with **(a) **AG1478 (1 μM) or PP1 (10 μM) and wortmannin (0.1 μM) or **(b) **PD98059 (50 μM) for one hour, followed by progesterone (P4) treatment at 30 ng/ml for 24 hours. Western blot analyses were performed with diverse anti-snail and/or epithelial-mesenchymal transition (EMT) relevant antibodies as indicated. The data are representative for three individual experiments.

To explore the intermediate pathways that regulate expression of snail/EMT proteins in the downstream of P4/mPRα signaling, several pathway specific inhibitors were tested in the current study. As shown in Figures 5a and 5b, the EGFR inhibitor (AG1478) and PI3K inhibitor (wortmannin) significantly blocked the P4-regulated snail/EMT protein expression in MB468 cells; while the ERK1/2 inhibitor (PD98059) did not block the P4's effect on snail/EMT. Additional file 6 showed that P4 induced phosphorylation of EGFR, Akt, Src, and ERK1/2; and coordinated pathway inhibitors repressed the P4-induced phosphorylation, indicating the functionality of these inhibitors. These results suggest that the signaling cascades of the P4-repressed snail/EMT in MB468 cells are mainly intermediated through the EGFR and PI3K/Akt pathways. However, the roles of Src family kinase inhibitor (PP1) in modulating EMT remain unclear as compared with that of other pathway inhibitors. PP1 did not block the P4's action on expression of snail and fibronectin, but it did block the P4's action on expression of occludin and E-cadherin.

**Figure 6:**
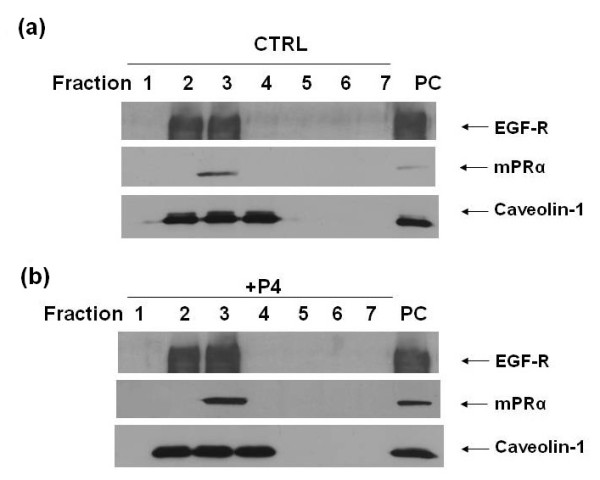
**Co-localization of mPRα and EGFR in caveolar membrane of MB468 cells**. Caveolar (Cav) fractions were isolated and immunoblotted for detecting Cav-1, membrane progesterone receptor (mPR) α, and epidermal growth factor receptor (EGFR) after the MB468 cells were treated **(a) **without and **(b) **with progesterone (P4) at 30 ng/ml for 24 hours. The Figures 6a and b show that Cav-1, mPRα, and EGFR are co-localized in the caveolar fraction #3 component regardless of P4 treatments. The results are representative for two separate experiments. PC, mPRα positive cells (HEK293).

As it has been reported that human BPBC cells often over express Cav-1, which is a major component of caveolae membrane and often negatively regulates the function of other caveolar-bound signaling molecules including EGFR [26-29]. To confirm the membrane location of mPRα and potential cross relation with other caveolae-bound signaling molecules, the caveolar fraction proteins were isolated from MB468 cells. As shown in Figures 6a and b, Cav-1 appeared in the fractions 2 to 4, suggesting these fractions mainly consist of caveolar membrane. Importantly, mPRα appeared in the fraction 3 where EGFR was also located, indicating the potential crosstalk between mPRα and EGFR.

### MPRα expression in human benign and malignant breasts

**Figure 7:**
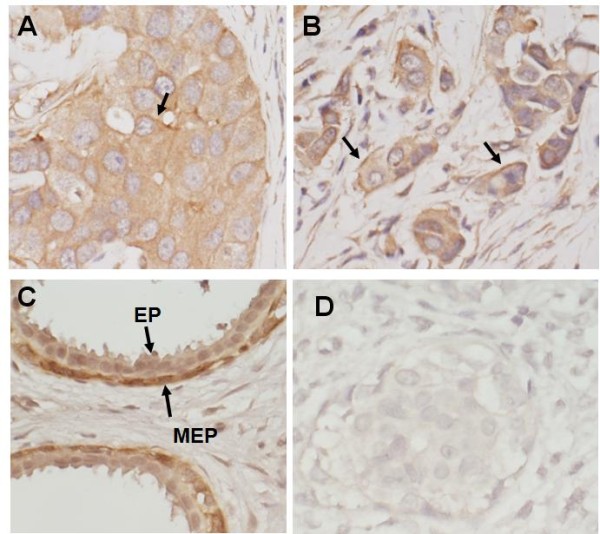
**Expression of mPRα in human breast cancer tissues**. **(a and b) **The low to intermediate intensities of membrane progesterone receptor (mPR) α stains in the cytoplasm of most human breast cancer cells. Apparent cytoplasm-membrane stains were observed in some of the cancer cells (black arrows). **(c) **The immunostain of mPRα in normal human breast. EP,ductal epithelium; MEP, myoepithelium.

To evaluate expression of mPRα in human breasts, tissue microarray slides were studied by immunohistochemistry. As shown in Table 1, 94 of 105 breast cancer tissues were stained positive for anti-mPRα. The positive signals are mainly observed in cytoplasm (Figure 7a) and/or cell membrane of cancer cells (Figure 7b). There were 14 triple-negative breast cancers (TNBC) among these breast cancer tissues. Most of these TNBC (13 of 14) were moderate to strong positive for mPRα stain. In addition, mPRα was also detected in all normal and/or benign breast tissues. The ductal and alveolar epithelial cells of breast were shown to be negative or weak positive while the myoepithelial cells were shown to be strong positive for mPRα (Figure 7c).

**Table 1:** Positivity of mPRα expression in human breast cancers

mPRα reactivity	TNBC	Non TNBC	Total
Positive (%)	13 (92.9)	81 (89)	94(89.5)
Negative	1	10	11
Total	14	91	105

No significant difference was found between the positive rates of mPRα (TNBC vs. non TNBC groups, *P *= 0.312, Fisher's exact test). MPRα, membrane progesterone receptor α; TNBC, triple-negative cancers.

## Discussion

Classically, the actions of P4 on breast cancer cells are attributed to the binding of nuclear PR and subsequent activation of the downstream target genes. Lange and colleagues proposed that P4 acts as a priming agent in breast cancer and, in his scenario, breast cells can be directed toward one path or another by crosstalking between the P4/PR complex and other signaling pathways [30]. In the PR-negative MB231 cells, P4 showed no effect on cell proliferation and invasion. However, after introducing exogenous PR cDNA into MB231 cells, the PR-expressing MB231 cells exhibited less proliferative activities after P4 treatment than the parental MB231 cells [31]. With this PR-expressing cell model, Sumida and colleagues demonstrated that P4 induced remarkable EMT-like changes in cell morphology and surface adhesion structures [31]. In the current study, we showed that P4 treatment *in vitro *inhibited EMT relevant proteins in the late passage MB468 cells. A negative association between P4 and snail expression was observed (Figure 1b). Consistent with down-regulation of snail expression, other EMT-relevant proteins, such as E-cadherin, occludin, and fibronectin, were subsequently modulated by P4 (Figure 1c); and these molecular changes were accompanied with cell morphological reversion from mesenchymal- to epithelial-like phenotypes (Figure 1d). Our results indicate that P4 functions as an anti-EMT hormone in MB468 cells *in vitro*. It is still unclear how P4 regulates these EMT events and what the cell mediators of P4 are.

The membrane progestin receptor, mPRα, has recently been identified as an intermediary factor of the progestin-induced intracellular signaling cascades in the PR-negative breast cancer cell lines *in vitro *[6,12]. The expression of mPRα in human breast cancer tissues, however, has not been well evaluated. With PCR assay, Dressing and Thomas reported expression of mPRα mRNA in both normal and malignant breast tissues [7]. Using an *in vitro *hormone binding technique and a FITC-conjugated BSA-progesterone, Pelekanou and colleagues detected the 'membrane-associate receptor for progesterone' in 57 of 61 breast cancers (94%) [32]. In this report, the protein expression of mPRα was detected in both human benign and malignant breasts (Table 1), which is quite consistent with Pelekanou's result. The receptor was also demonstrated in all but one triple-negative breast cancer - a type of cancer that shares many common features with BPBC [33,34]. Moreover, in the benign breasts, strong positive stain for mPRα was detected in the basal myoepithelial cells. Recently we showed that the mammary ducts of normal mice were positive for both PR and mPRα. The PR was predominantly seen in the ductal epithelium, while mPRα was mostly observed in the basal myoepithelial cells [22]. The synergistic roles of mPRα and PR in normal mammary glands remain to be explored.

The mPRα receptor has been associated with many physiologic functions in vertebrates. It induces oocyte maturation, stimulates sperm hypermotility, down-regulates GnRH secretion, modulates T cell functions, and adjusts human myometrial cell contractility [6,7,11-13,35]. In agreement with the earlier studies performed in human myometrial cells and fish oocytes [12,13], we found that P4 up-regulated the expression of mPRα in MB468 cells (Figure 4a). Importantly, P4's actions on expression of snail/EMT-relevant proteins were significantly blocked by the mPRα specific siRNA (Figure 4c). In contrast, P4 treatment alone had no effect on snail expression in the parent MB231 cells, in which mPRα protein is undetectable by western blot assay (Figure 4b). We thought that the exogenous mPRα cDNA stable transfection would cause the cell EMT responding to the P4 treatment. Unexpectedly, the expression of snail/EMT relevant markers remained unchanged after P4 treatments, indicating other factors in the P4/mPRα signaling pathway were still blocked.

The mesenchymal phenotype of MB231 cells under normoxic culture conditions has been associated with high levels of urokinase-type plasminogen activator (uPA) and uPA receptor (uPAR) expression and silencing uPA expression decreased expression of vimentin and snail and induced epithelial-like transition in the cells [16]. In the current study, we showed that the P4 repressed EMT in MB231 cells is correlated to the mutant *pten *and activation of PI3K/Akt signaling pathway. PTEN is a major inhibitor of the PI3K/Akt signaling pathway. Loss of PTEN protein expression occurs commonly in breast cancer, which has been associated with loss of ER [36] and resistance to cancer therapies [37]. The PTEN-deficient cell lines displayed greater sensitivity to the growth inhibitory effects of the PI3K inhibitor, LY294002, as compared with the PTEN-positive cell lines [38]. Recently major differences have been reported in the status of PI3K/Akt pathway and function of PTEN between MB468 and MB231 cells [15,39]. It was assumed that the activation of PI3K/Akt pathway, resulting from a dysfunctional PTEN, is essential for the P4-repressed EMT. In further study, we demonstrated that the expression of snail/EMT-relevant proteins in the mPRα expressing MB231 cells was significantly modulated after incubating the cells with P4 plus PTEN inhibitor - bpV(phen) (Figure 4d). However, activation of PI3K/Akt seems not to be essential for the P4-repressed cell proliferation because the growth reduction of the mPRα-expressing MB231 cells could be induced by P4 treatment alone. It is assumed that the P4 inhibited cell proliferation may go through other pathways, such as the secondary messenger pathway through activation of pertussis toxin-sensitive inhibitory G proteins and MAPK/Erk1/2 [12,20].

**Figure 8:**
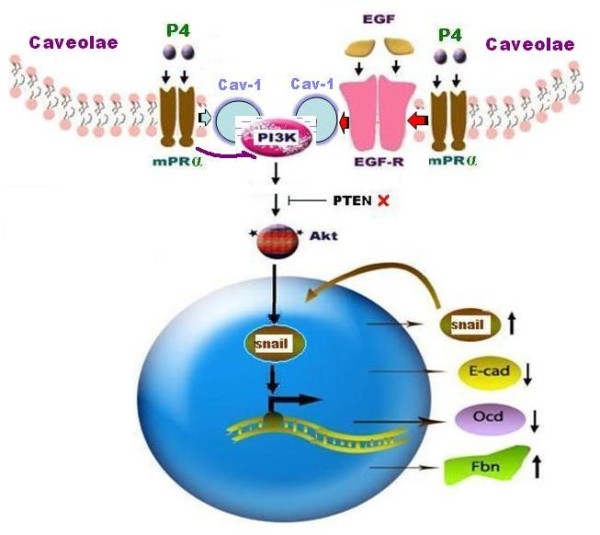
**Schematic illustration of molecular pathway initiated by P4 signaling in BPBC cells**. MB468 cells usually maintain the activities of PI3K/Akt at high levels because these cells contain a mutant *pten *gene and over expressed epidermal growth factor receptor (EGFR). Activation of PI3K/Akt pathway is a vital signal for cell survival and epithelial to mesenchymal transition (EMT). It is assumed that the binding of progesterone (P4) to membrane progesterone receptor (mPR) α in the caveolar membrane of the cells inhibits EMT-relevant events either directly through Caveolar (Cav) 1 activation or through a cross interaction with EGFR and trans-activation of Cav-1 which subsequently inactivate PI3K/Akt pathway. Inactivation of PI3K/Akt pathway subsequently inhibits the nuclear translocation of snail and then modulates expression of other EMT-relevant proteins.

When exploring the intermediate pathways that regulate snail/EMT in P4 signaling, we showed that P4's actions on EMT were significantly blocked in the late passage MB468 cells by AG1478 (an EGFR inhibitor) and wortmannin (PI3K inhibitor), suggesting EGFR and PI3K/Akt pathways are involved in the P4 repressed EMT events. Studies have shown that along with other signaling molecules such as PDGFR, Ha-ras, and c-Src, both EGFR and PI3K are distributed in the caveolar vesicles in which Cav-1 serves as a main structure component [40]. Cav-1 usually functions as a negative regulator of other caveolar-bound signaling molecules [26-29]. Existing data has shown that BPBC is associated with high expression of Cav-1 [41,42] and EMT of cancer cells is dependent upon the presence of Cav-1 [40]. Okamoto and colleagues showed that long-term EGF treatment reduced expression of Cav-1 in cancer cells; and subsequently up-regulated snail and down-regulated E-cadherin expression [40]. Lu and colleagues demonstrated that EGF treatment of human tumor cells that over express EGFR caused a dramatic alteration in cell-cell contacts and internalization of E-cadherin [43]. It was assumed that upon binding to EGF, EGFR forms homodimers or heterodimers which result in the activation of their intrinsic kinases and autophosphorylation of specific tyrosine residues within their cytoplasmic domains [44]. The activated EGFR may recruit other molecular signaling complexes such as PI3K, via several potential paths. For example, EGFR may bind to and recruit PI3K directly because the canonical binding sites for the regulatory subunit of PI3K are not found on EGFR [45]; it may also employ the docking protein Gab1 to recruit PI3K [46]. In addition, the EGFR adapter (Shc) may recruit PI3K by assembly of a Shc-Grb2-Gab2-PI3K complex [47]. The role of PI3K/Akt pathway in cancer EMT has been well documented in various human malignancies [48-50]. The proposed mPRα dependent molecular pathways that inhibit EMT of BPBC are schematically illustrated in Figure 8.

The essential roles of c-Src pathway in the P4/PR signaling pathways have been demonstrated in human breast cancer cells that is T47 D cells. The cell anchorage-independent growth was stimulated by progestin and blocked by inhibition of Erk1/2, c-Src, EGFR, or RNA interference of Wnt-1 [51]. Recently Lester and colleagues reported that when MB468 breast cancer cells were cultured in a hypoxia condition expression of uPAR was increased, cell-cell junctions were disrupted, vimentin expression was increased, and E-cadherin was lost from cell surfaces, indicating enhancement of EMT [16,52]. Lester and colleagues proposed a model in which Src family kinases may concert with other cell signaling factors, including PI3K and ERK1/2 and play an essential role in the regulation of uPA and uPAR and EMT [16]. In this report, we found that in the late passage MB468 cells, the Src family kinases inhibitor (PP1) did not block the P4's action on snail and fibronectin (one of the mesenchymal phenotypes), but it blocked the P4's action on expression of occludin and E-cadherin (epithelial phenotypes). The roles of Src family kinases on the P4-repressed EMT remain to be explored.

## Conclusions

In summary, using two human BPBC cell lines as models, we identified a PR-independent pathway that involves the signaling cascade of EMT through a caveolae-bound signaling complex namely mPRα, Cav-1, EGFR, and PI3K/Akt. It is assumed that mPRα receptor is the key modulator of EMT located on the caveolar membrane of BPBC cells. Through the receptor-mediated mechanisms, P4 directly inactivates the PI3K-snail-EMT pathway or interacts with Cav-1 and modulates the activities of the EGFR pathway, which then cross inhibit PI3K pathway, and eventually suppresses the cell EMT. The proposed pathway is attractive for further understanding the molecular mechanisms of EMT and for developing novel therapeutic strategies against BPBC.

## Abbreviations

BPBC: basal phenotype breast cancers; Cav-1: caveolar-1; DMEM: Dulbecco's modified Eagle's medium; E2: estrogen; EGF: epidermal growth factor; EGFR: epidermal growth factor receptor; EMT: epithelial-mesenchymal transition; ER: estrogen receptor; FBS: fetal bovine serum; HEK: human embryonic kidney; HER: human epidermal growth factor receptor; MB231: MDA-MB231 cells; MB468: MDA-MB468 cells; MES: 2-(*N*-morpholino) ethanesulfonic acid; mPRα: membrane progesterone receptor α; P4: progesterone; PBS: phosphate-buffered saline; PCR: polymerase chain reaction; PR: progesterone receptor; PTEN: phosphatase and tensin homolog gene; siRNA: small interferring RNA; TNBC: triple-negative breast cancers; uPA: urokinase-type plasminogen activator; uPAR: uPA receptor.

## Competing interests

The authors declare that they have no competing interests.

## Authors' contributions

LZ functioned as a main researcher in this study and her work covered most of the assays. WL functioned as a researcher in this study whose contribution was mainly focused on some of the protein expression. SY proposed the hypothesis and designed the study, functioned as the supervisor and PI, and composed the manuscript.

## Supplementary Material

Additional file 1 **Morphology of early and late passage MB468 cells**. The cultured MB468 cells at early passages (6 passages) appeared as oval and/or polygonal shapes; and after multiple passages (50+ passages), these cells exhibit apparent mesenchymal phenotypes with spindle and elongated shapes as indicated. Photos (DIC images) were taken by confocal microscopy at 200× magnification. Click here for file

Additional file 2 **Diverse intensities of mPRα immunostains in human breast cancers**. **(a) **Strong positive stain - most of the cancer cells are stained dark brown. **(b) **Modulate positive - most of the cancer cells are stained modulate brown. **(c) **Weak positive - light brown. **(d) **Negative stain - very light brown or no stain. mPRα, membrane progesterone receptor α. Click here for file

Additional file 3 **Cell morphology of late passage MB468 cells at low magnification with/without P4 treatment**. Photos (DIC images) were taken by confocal microscopy at 200× magnification. This is an enlarged view of the Figure 1d. P4, progesterone. Click here for file

Additional file 4 **Dose curve of the P4-repressed cell proliferation of MB 468 cells**. The growth-arrested MB468 cells were treated with different doses of progesterone (P4) as indicated. The cell proliferation was inhibited in a dose-dependent manner (15 ng/ml - 20%, 30 ng/ml - 25%, 60 ng/ml - 48%). The data were averaged from three experiments and the graph represents an averaged data expressed as fold change over basal. * *P *< 0.05 for difference of cell proliferation induced by P4 vs. vehicle alone. Click here for file

Additional file 5 **Knocking down expression of mPRα by siRNA in MB468 cells**. MB468 cells were transfected with indicated amount of membrane progesterone receptor α (mPRα) siRNA. Western blot analysis was performed with anti-mPRα and anti-α-tubulin antibodies. As shown in the figure, more than 90% of mPRα expression was inhibited by transfection of mPRα siRNA at 50 nM. The data are representative for three experiments. Click here for file

Additional file 6 **Treatment of MB468 cells with P4 alone**. A figure showing that the treatment of MB468 cells with progesterone (P4) alone significantly promotes phosphorylation of **(a) **epidermal growth factor receptor (EGFR), **(b) **Akt, **(c) **Src and **(d) **ERK1/2**; **and co-treatment of the cells with P4 and the specific pathway inhibitors abolishes the P4-induced phosphorylation on diverse pathway components (i.e. EGFR, Akt, Src and ERK1/2), indicating the effectiveness of P4 treatments in the activation of diverse molecular pathways. Click here for file
