# Supplementary figures and images for: Progesterone reverses the mesenchymal phenotypes of basal phenotype breast cancer cells via a membrane progesterone receptor mediated pathway
Source: Breast Cancer Res. 2010 Jun 11;12(3):R34. doi: 10.1186/bcr2588 (PMC2917029; doi:10.1186/bcr2588)

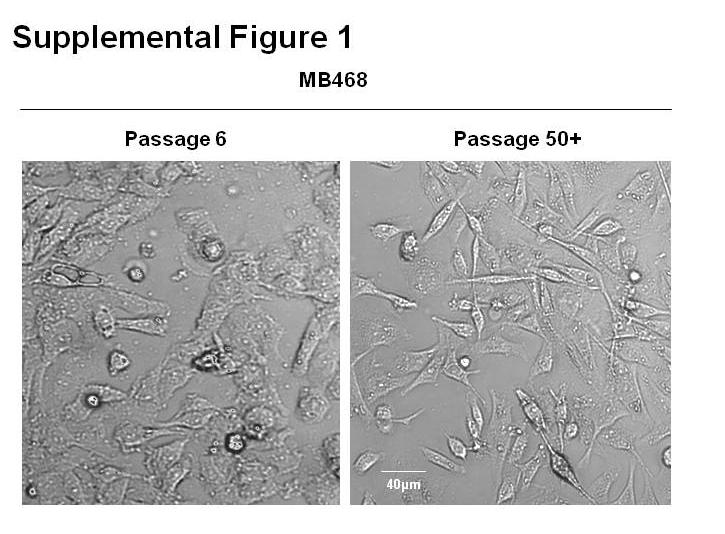

Supplement: Additional file 1 — Morphology of early and late passage MB468 cells. The cultured MB468 cells at early passages (6 passages) appeared as oval and/or polygonal shapes; and after multiple passages (50+ passages), these cells exhibit apparent mesenchymal phenotypes with spindle and elongated shapes as indicated. Photos (DIC images) were taken by confocal microscopy at 200× magnification. [file bcr2588-S1.JPEG]

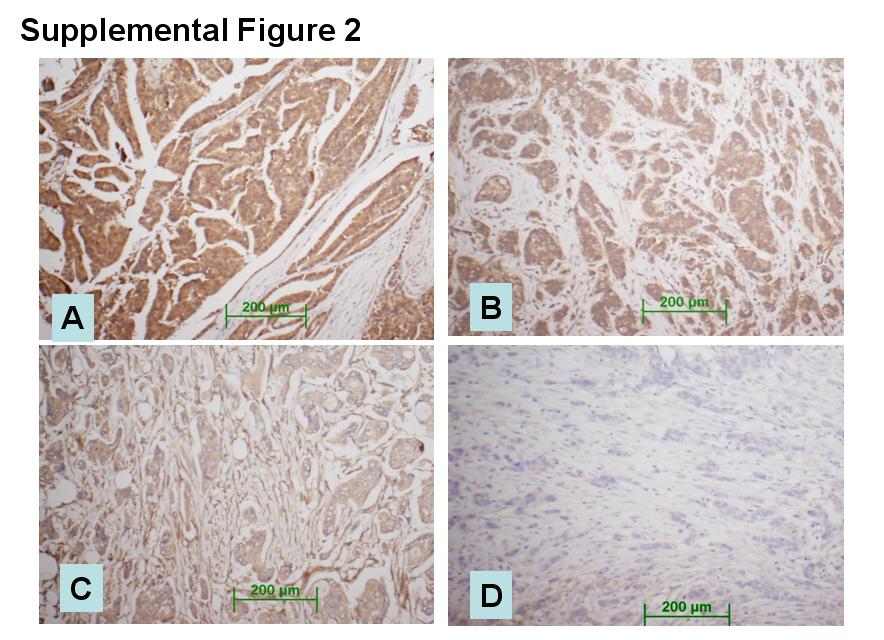

Supplement: Additional file 2 — Diverse intensities of mPRα immunostains in human breast cancers. (a) Strong positive stain - most of the cancer cells are stained dark brown. (b) Modulate positive - most of the cancer cells are stained modulate brown. (c) Weak positive - light brown. (d) Negative stain - very light brown or no stain. mPRα, membrane progesterone receptor α. [file bcr2588-S2.JPEG]

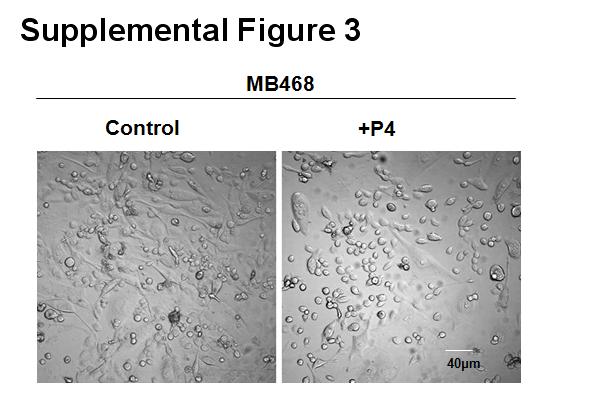

Supplement: Additional file 3 — Cell morphology of late passage MB468 cells at low magnification with/without P4 treatment. Photos (DIC images) were taken by confocal microscopy at 200× magnification. This is an enlarged view of the Figure 1d. P4, progesterone. [file bcr2588-S3.JPEG]

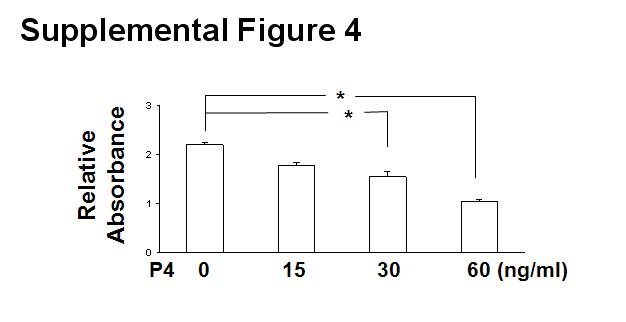

Supplement: Additional file 4 — Dose curve of the P4-repressed cell proliferation of MB 468 cells. The growth-arrested MB468 cells were treated with different doses of progesterone (P4) as indicated. The cell proliferation was inhibited in a dose-dependent manner (15 ng/ml - 20%, 30 ng/ml - 25%, 60 ng/ml - 48%). The data were averaged from three experiments and the graph represents an averaged data expressed as fold change over basal. * P < 0.05 for difference of cell proliferation induced by P4 vs. vehicle alone. [file bcr2588-S4.JPEG]

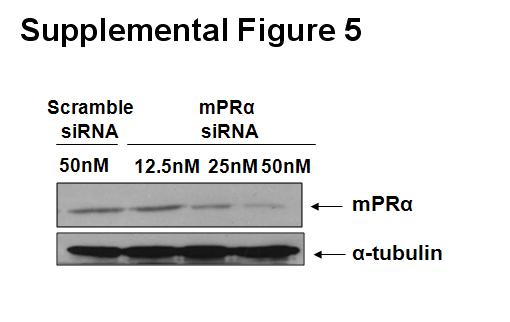

Supplement: Additional file 5 — Knocking down expression of mPRα by siRNA in MB468 cells. MB468 cells were transfected with indicated amount of membrane progesterone receptor α (mPRα) siRNA. Western blot analysis was performed with anti-mPRα and anti-α-tubulin antibodies. As shown in the figure, more than 90% of mPRα expression was inhibited by transfection of mPRα siRNA at 50 nM. The data are representative for three experiments. [file bcr2588-S5.JPEG]

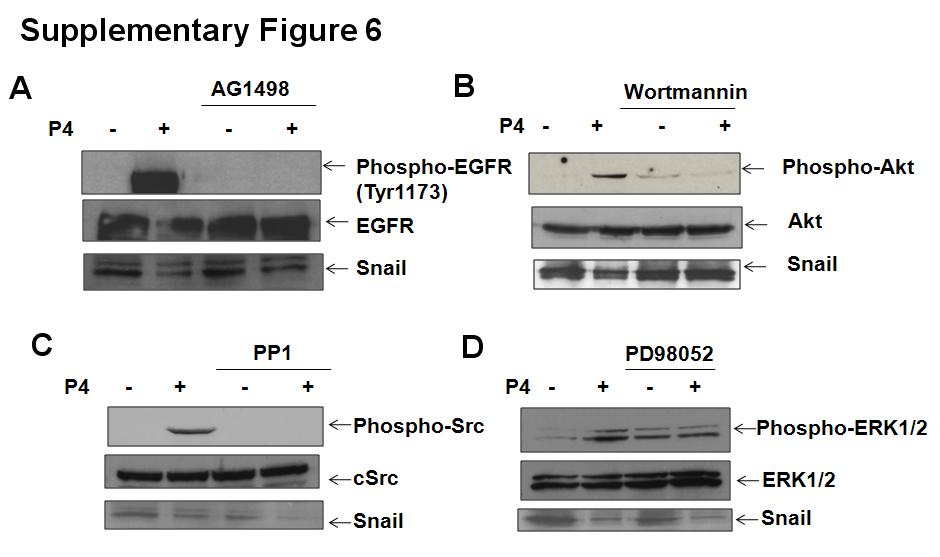

Supplement: Additional file 6 — Treatment of MB468 cells with P4 alone. A figure showing that the treatment of MB468 cells with progesterone (P4) alone significantly promotes phosphorylation of (a) epidermal growth factor receptor (EGFR), (b) Akt, (c) Src and (d) ERK1/2; and co-treatment of the cells with P4 and the specific pathway inhibitors abolishes the P4-induced phosphorylation on diverse pathway components (i.e. EGFR, Akt, Src and ERK1/2), indicating the effectiveness of P4 treatments in the activation of diverse molecular pathways. [file bcr2588-S6.JPEG]
